# Structural Characteristics and Anticancer Activity of Fucoidan from the Brown Alga *Sargassum mcclurei*

**DOI:** 10.3390/md11051456

**Published:** 2013-05-06

**Authors:** Pham Duc Thinh, Roza V. Menshova, Svetlana P. Ermakova, Stanislav D. Anastyuk, Bui Minh Ly, Tatiana N. Zvyagintseva

**Affiliations:** 1 Nhatrang Institute of Technology Research and Application, Vietnam Academy of Science and Technology, 02 Hung Vuong Street, Nhatrang 650000, Socialist Republic of Vietnam; E-Mails: ducthinh.nitra@gmail.com (P.D.T.); bminhly@nitra.ac.vn (B.M.L.); 2 Laboratory of Enzyme Chemistry, G.B. Elyakov Pacific Institute of Bioorganic Chemistry, Far Eastern Branch, Russian Academy of Sciences, 159 100-Let Vladivostoku Ave., Vladivostok 690022, Russian Federation; E-Mails: swetlana_e@mail.ru (S.P.E.); sanastyuk@piboc.dvo.ru (S.D.A.); zvyag@piboc.dvo.ru (T.N.Z.)

**Keywords:** brown alga, *Sargassum*, fucoidan, oligosaccharides, structure, anticancer activity

## Abstract

Three different fucoidan fractions were isolated and purified from the brown alga, *Sargassum mcclurei*. The **SmF1** and **SmF2** fucoidans are sulfated heteropolysaccharides that contain fucose, galactose, mannose, xylose and glucose. The **SmF3** fucoidan is highly sulfated (35%) galactofucan, and the main chain of the polysaccharide contains a →3)-α-l-Fuc*p*(2,4SO_3_^−^)-(1→3)-α-l-Fuc*p*(2,4SO_3_^−^)-(1→ motif with 1,4-linked 3-sulfated α-l-Fuc*p* inserts and 6-linked galactose on reducing end. Possible branching points include the 1,2,6- or 1,3,6-linked galactose and/or 1,3,4-linked fucose residues that could be glycosylated with terminal β-d-Gal*p* residues or chains of alternating sulfated 1,3-linked α-l-Fuc*p* and 1,4-linked β-d-Gal*p* residues, which have been identified in galactofucans for the first time. Both α-l-Fuc*p* and β-d-Gal*p* residues are sulfated at C-2 and/or C-4 (and some C-6 of β-d-Gal*p*) and potentially the C-3 of terminal β-d-Gal*p*, 1,4-linked β-d-Gal*p* and 1,4-linked α-l-Fuc*p* residues. All fucoidans fractions were less cytotoxic and displayed colony formation inhibition in colon cancer DLD-1 cells. Therefore, these fucoidan fractions are potential antitumor agents.

## 1. Introduction

Brown algae are a rich and easily renewable source of biologically active polysaccharides, including alginic acids, laminarans and fucoidans. These unique compounds are very interesting due to their wide spectrum of pharmacological properties and low toxicity *in vivo*. Alginic acids are the most studied polysaccharides from brown algae because they have been successfully used over time in the medical and pharmaceutical industries, among others. Other valuable polysaccharides, such as laminarans and fucoidans, have not been well studied compared to alginic acids. Fucoidans have a complex and non-regular structure, making a detailed structural analysis of these molecules difficult. The structural diversity of fucoidans is still not fully characterized.

Recently, collisionally induced dissociation tandem ESI mass spectrometry (CID ESIMS/MS) and potential lift tandem MALDI-TOF mass spectrometry (LIFT-TOF/TOF) were successfully applied as complementary tools for the study of fucoidans. Using tandem mass spectrometry and autohydrolysis as an alternative strategy for fucoidan depolymerization, it was reported that the composition and structural features of LMW-components were specific and dependent on the linkage and sulfated pattern of native polysaccharides [1,2].

Based on known data, fucoidans are members of a family of sulfated homo- and heteropolysaccharides that are mainly comprised of α-l-fucopyranose residues. In addition, fucoidans can contain small amounts of other monosaccharide residues, including galactose, xylose, mannose, ramnose and uronic acids. Acetyl groups are also known to be constituents of fucoidans. It has been established that fucoidans have multiple biological activities [3,4].

Brown algae of the *Sargassum* genus belong to the family Sargassaceae of the order Fucales, and these algae are widely distributed on the coast of Europe, Asia, Africa, Australia, North America and South America. Fucoidans isolated from these algae have various structural characteristics, ranging from sulfated homofucans to heteropolysaccharides [5,6,7,8].

Only a few groups have reported the isolation of nearly pure fucans. Three fractions of polysaccharides were isolated from *S. horneri *(Republic of Korea, May 2009): sulfated fucan (14.9% sulfate content), nonsulfated fucan and sulfated rhamnofucan (16.9% sulfate content) [6]. Highly branched sulfated fucan (17% sulfate content) was isolated from the *S. horneri *(Japan, May 1999), which revealed the presence of various linkages, such as 1,2-, 1,3- and 1,4-linkages between monosaccharide residues [8]. Sulfate groups were identified at C-2 or C-4 position in the 1,3-linked residue and at the C-3 position in the 1,2- and 1,4-linked residues. In addition, disulfated 1,4-linked residues were present.

A fraction containing sulfated galactofucan was isolated from *S. stenophyllum *(Brazil, February 1996), and it contained small amounts of glucuronic acid and a high percentage of sulfate groups, which were found to be concentrated on fucose residues. The sulfate contents of all fucoidan fractions ranged from 19.0% to 28.3% [9]. A fucoidan containing fucose (79.1 mol%) and galactose (19.9 mol%), with xylose and glucose impurities, was isolated from *S. trichophyllum* (Japan, May 2006). The sulfate content of this fucoidan was 25.5%. Methylation analysis revealed that this fucoidan was mainly composed of terminal, 1,4- and 1,3-linked fucose and terminal, 1,2- and 1,6-linked galactose residues [10]. The previous investigation revealed that the fucoidan fractions from some Vietnamese *Sargassum *species (*S. polycystum*, *S. oligocystum*, *S. mcclurei*, *S. swartzii *and *S. denticaprum*) were sulfated fucogalactans that contained a minor amount of other sugars and uronic acids [11].

Heteropolysaccharides isolated from *Sargassum* were composed of fucose, galactose, glucose, mannose, xylose, glucose and glucuronic acid, and their sulfate content ranged from 6.2% to 28.8% [5,7,12]. The polysaccharide compositions of algae of the genus *Sargassum *may have differed because the specimens were collected at different locations and life stages [13,14,15] and because different extraction methods were used [16].

There are some data regarding the biological effects of fucoidans isolated from of *Sargassum* genus algae. These fucoidans are of particular interest because these compounds display the following broad spectrum of biological activities: anticoagulant, antiviral, antiproliferative, antioxidant, and anticancer [5,6,7,8,10,11,12,17]. Colorectal cancer is one of the most common cancers, and it has a high propensity to metastasize [18]. It was reported that fucoidans induce apoptosis in several colon cancer cell lines [19,20]. However, most previous studies have used a commercially available fucoidan from *Fucus vesiculosus*, which contains more than 16 different sulfated polysaccharides with varying proportions of the individual monosaccharide residues [21]. In our previous investigations, we showed that fucoidans from some Vietnamese *Sargassum *species, *Eisenia bicyclis* and *Saccharina cichorioides*, were nontoxic towards colon cancer cells and inhibited colony formation [22,23,24]. In this report, we study the anticancer activity of fucoidans from the brown alga, *Sargassum mcclurei* Setchell, 1933 (Socialist Republic of Vietnam), to find structural characteristics that are important for its anticancer activity. Bioactive compounds derived from natural sources (such as fucoidans from brown algae) with potential anticancer activity could be very important as food supplements to prevent colorectal cancer.

## 2. Results and Discussion

### 2.1. Isolation and Characterization of Three Fucoidan Fractions from *S. mcclurei*

Water-soluble polysaccharides were extracted from the dry, defatted brown alga, *S. mcclurei* (**Sm**), using 0.1 M HCl. Laminaran (**SmL**) and crude fucoidan (**SmF**) were isolated from the concentrated and neutralized extract by hydrophobic chromatography on Polychrome-1. The yield of laminaran and crude fucoidan were 0.02% and 2.7% of the dried, defatted alga weight, respectively. Crude fucoidan was fractionated by anion-exchange chromatography on Macro-Prep DEAE eluting with NaCl gradients ranging from 0.1 to 2 M (supplementary data). Three fractions, **SmF1**, **SmF2** and **SmF3**, were obtained. The yields and composition of the purified fucoidans are listed in Table 1.

**Table 1:** Yields and monosaccharide composition of fucoidan fractions from *S. mcclurei *obtained by anion-exchange chromatography (Macro-Prep DEAE).

Fucoidan	Eluent [NaCl], M	Yield, % *	Sugar total, % **	SO_3_Na, % **	Monosaccharide composition, mol%				
					Fuc	Man	Gal	Xyl	Glc
**SmF1**	0.7–0.8	8.4	44.6	16.8	27.2	34.0	19.6	6.4	12.8
**SmF2**	0.8–1.4	18.2	64.6	25.7	44.8	5.4	34.1	5.3	10.4
**SmF3**	1.4–1.8	10.5	53.2	35.0	58.5	0	41.5	0	0

* % of crude fucoidan weight; ** % of sample weight.

An analysis of the monosaccharide composition of these fractions indicated that **SmF1** and **SmF2** contained different amounts of fucose, galactose, mannose, xylose and glucose. The **SmF3** fucoidan was a highly sulfated galactofucan (35% sulfate content). All fucoidan fractions lacked proteins and polyphenols.

### 2.2. Structural Characteristics of the Fucoidan SmF3

The fucoidan fraction **SmF3** consisted of only fucose and galactose residues, and it had the highest sulfate content among all fractions (Table 1). Therefore, it was used for structural elucidation.

Like many native algal fucoidans, the fucoidan **SmF3** had very complex ^13^C NMR spectrum (supplementary data) that was difficult to completely elucidate. The ^13^C NMR spectrum of **SmF3** exhibited several major signals, most of which showed a certain degree of multiplicity. This observation suggested diversity in the positions of glycosidic linkages and/or multiplicity in the sulfation patterns. The ^13^C NMR spectrum of the native fucoidan **SmF3** contained several intense signals in the anomeric (96.0–100.4 ppm) and the high-field (15.5–17.7 ppm) regions, which are typical of the C-1 and C-6 carbons of α-l-fucopyranosides. In addition, signals at 61.7 and 67.0 ppm were attributed to the free and glycosylated C-6 carbon of β-d-galactose residues, respectively, and signals at 102.7–104.1 ppm were attributed to the C-1 of β-d-galactose residues. Signals corresponding to acetate groups and uronic acids were absent.

The desulfation of **SmF3** was performed to simplify the structure of this fucoidan. The ^13^C NMR spectrum of desulfated fucoidan **SmF3-DS** (supplementary material) contained a large group of signals in the anomeric region (96–104 ppm). Thus, the fucoidan **SmF3** contained several types of fucose and galactose residues that differed in the type of linkage. The intensity of a peak at 61.7 ppm, corresponding to the free C-6 carbon of galactose residues, increased significantly after desulfation, indicating that some galactose residues contained sulfate groups at C-6.

The desulfated **SmF3-DS** fucoidan was methylated with methyl iodide in the presence of sodium hydroxide in DMSO [25]. The methylated polysaccharide was hydrolyzed, and the resulting mixture of partially methylated monosaccharides was analyzed as alditol acetates by GLC-MS [26] (Table 2).

**Table 2:** Methylation analysis of desulfated fucoidan **SmF3-DS**.

Partially methylated fucitol or galactitol acetates	mol%	Linkage type
2,3,4-tri-*O*-methyl-fucitol	5	Fuc1→
2,3-di-*O*-methyl-fucitol	12	→4Fuc1→
2,4-di-O-methyl-fucitol	46	→3Fuc1→
2,3,4,6-tetra-*O*-methyl-galactitol	8	Gal1→
2,3,6-tri-*O*-methyl-galactitol	18	→4Gal1→
2,3,4-tri-*O*-methyl-galactitol	2	→6Gal1→
3,4-di-*O*-methyl-galactitol	1	→2,6Gal1→
2,4-di-*O*-methyl-galactitol	2	→3,6Gal1→
1,2,3,4-tetra-*O*-methyl-galactitol	6	→6Gal

The fucoidan **SmF3** contained mainly 1,3-linked fucose, less 1,4-linked galactose, 1,4-linked fucose and 1,6-linked galactose residues. The fucoidan **SmF3** contained fucose and galactose on its non-reducing end, and it contained only 6-linked galactose on the reducing end. The detection of 3,4- and 2,4-di-*O*-methyl-galactitol suggested the presence of side chains and/or residual sulfates in **SmF3**.

### 2.3. Mass Spectrometric Analysis of the Oligosaccharides Obtained by the Autohydrolysis of the Fucoidan SmF3

For a detailed study of the structural characteristics of the fucoidan **SmF3**, a mass spectrometric analysis of oligosaccharides was obtained by the autohydrolysis of the native polysaccharide. The low molecular weight (LMW) fraction, **SmF3-AH** (supernatant), was isolated from the mixture that was obtained after autohydrolysis by the addition of aqueous ethanol (Section 2.3.8). We obtained 85% yield of **SmF3-AH** from **SmF3**. The relative amount of galactose in its monosaccharide composition (Fuc:Gal ≈ 2:1) *versus* polysaccharide **SmF3** (Fuc:Gal ≈ 3:2) increased. The observation of such a high amount of galactose in the autohydrolysis mixture suggested a different structural role for galactose in this fucoidan compared to the fucoidans from Laminariales, in which this monosaccharide can form prolonged chains [13,27,28]. In addition, the amount of galactose in the LMW fractions obtained from autohydrolysates of galactofucans from *Laminaria gurjanovae* [29] and *Costaria costata* [13] did not exceed 10 mol%. However, in the case of Laminariales, the pellet (which was resistant to autohydrolysis fraction) contained high amounts of galactose. An analysis of the monosaccharide composition of the pellet (Section 2.3.8) obtained from **SmF3** displayed reduced galactose and increased fucose levels. These data suggest that the fucoidan **SmF3** did not contain prolonged blocks of built up of galactose, which was confirmed by the analysis of short fragments using negative-ion tandem MS.

**Figure 1:**
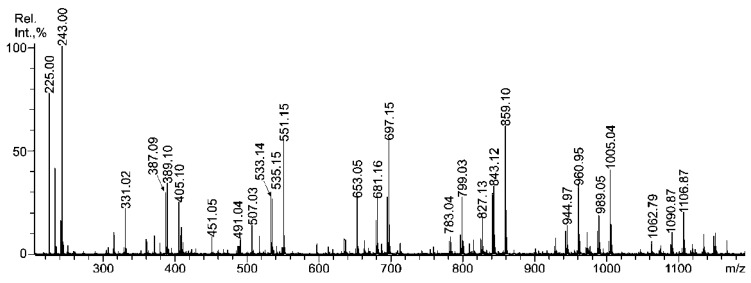
Negative-ion MALDI-TOFMS of LWM oligosaccharide fraction **SmF3-AH**, obtained from a fucoidan of *S. mcclurei* by autohydrolysis.

MALDI-TOFMS (Figure 1) of the autohydrolysis mixture was easier to interpret than the ESIMS analysis (not shown).

According to the resultsof both methods, the main component of the mixture was monosulfated fucose, at *m/z* 243.0. The fucoidan **SmF3** was found to be highly sulfated (35%), and no disulfated fucose residue was detected in the oligosaccharide mixture using either method, in contrast to the highly sulfated fucan from *Saccharina cichorioides* (ex name *Laminaria*) [2]. However, both the ESIMS and MALDIMS spectra contained a set of intensive fragment ions (*m/z* 224.98, 387.09, 533.14, 695.15 and others) and large amounts of monosulfated fucooligosaccharides (*m/z* 389.10, 535.15, 681.16 and 827.13; see Table 2 for details). Excess fragmentation followed by desulfation was also observed during oligosaccharide, derived from 2,4-disulfated 3-linked α-l-fucan from *S. cichorioides* [2]. The presence of 3-linked α-l-Fuc*p* residues was supported by methylation analysis (Table 2). The results of mass spectrometric analysis revealed that the oligosaccharide content (obtained in the same conditions by autohydrolysis) of fucan with the main chain, which was composed of alternating α-(1→3)-/α-(1→4)-linked and less-sulfated Fuc*p *residues were different. It was shown that α-(1→4)-linkage type hydrolyzed more slowly [1,30]. The behavior of the autohydrolysis mixture components in ESI and MALDI ion sources differed slightly. Multisulfated ions (up to 3 sulfates per molecule) survived in the ESIMS more readily than in MALDI, which was able to detect only the disulfated species. In contrast, heavily sulfated (up to 5 sulfates per molecule) oligosaccharides derived from the fucan sulfate of the brown alga *F. evanescens* were readily detected using MALDI-TOFMS [1]. Thus, this excess desulfation was likely the effect of the structural features of the components rather than an artifact of the mass spectrometric method. However, it is known that MALDI-TOFMS works better for the analysis of complex mixtures [31]. It is possible that the fucoidan **SmF3** contains both highly sulfated galactose and fucose residues that are arranged spatially close to sulfate groups, which promotes excess fragmentation/desulfation during autohydrolysis and/or in the ion source of the mass spectrometer. The discussion on this matter will proceed further.

The structural features of some interesting ions were elucidated using the negative-ion tandem MS mode with both ESIMS and MALDIMS, using observations from previous studies on oligosaccharides derived from carrageenans, glucosaminoglycans and fucoidans. It was reported that the product ion spectra of [M − Na]^−^ featured an extensive series of B- and C-type (according to the nomenclature, suggested by Domon and Costello [32]) glycosidic cleavages, whereas the Y-type cleavage occurred mainly at the sulfated residues [33]. This glycosidic cleavage observation is consistent with the previously proposed [34] sulfate-mediated hydrogen transfer for B_1_-type ion formation. This mechanism justifies the observation that if the sulfate group of an oligosaccharide is spatially closer to the glycosidic linkage, it undergoes an easier B_1_-type fragmentation. ESIMS/MS and computational chemistry methods on 2- and 4-sulfated fucose residues [35] recently revealed that the formation of ^0,2^X/^0,2^A fragment ions required an unsubstituted proton on the C-3 hydroxyl group. Thus, no cross-ring cleavages could occur when 3-linked [2,13] and/or 3-substituted oligosaccharides were fragmented using CID ESIMS/MS or MALDI-TOFMS/MS.

**Figure 2:**
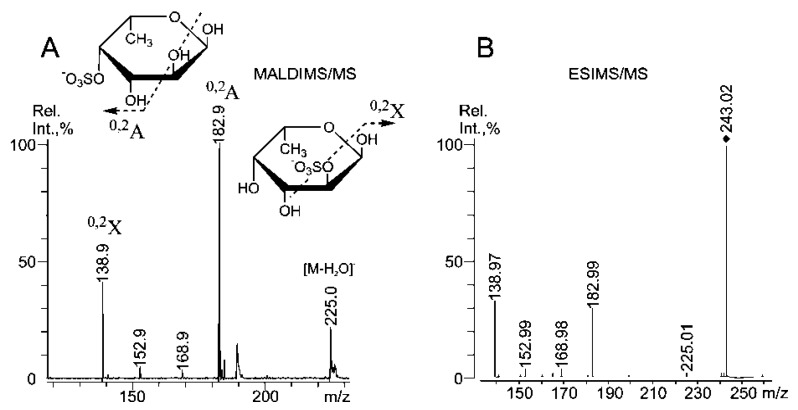
Negative-ion ESIMS/MS of [FucSO_3_]^−^ at *m/z* 243.02 (**A**), MALDIMS/MS of [FucSO_3_]^−^ at *m/z* 243.0 (**B**).

Negative-ion ESIMS/MS [GalSO_3_]^−^ at *m/z* 259.02 (supplementary data) identified fragment ions of equal intensity that are characteristic of sulfation at C-2 at *m/z* 138.98 (^0,2^X) and C-4 at *m/z* 198.99 (^0,2^A). A relatively high-intensity satellite signal from the ion at *m/z* 180.98 (^0,2^X-H_2_O) was observed upon the fragmentation of the 4-sulfated galactose residue (observed with ESIMS^3^). Thus, Gal residues are likely sulfated at C-2 or C-4 [36]. Sulfation at C-6 is also possible, as a relative increase in the free C-6 of Gal residues was detected by ^13^C NMR (see above) in the desulfated fucoidan **SmF3-DS**. The negative-ion ESIMS/MS and MALDI-TOFMS/MS (Figure 2A,B) analysis of monosulfated fucose [FucSO_3_]^−^ at *m/z* 243.02 was similar to that observed for *S. cichorioides*, where signals from C-4 (*m/z* 182.99) and C-2 (*m/z* 138.97) sulfation of α-l-Fuc*p* residues [35] were equal in ESIMS [2].

The loss of 4-sulfated fucose residues using the MALDI ion source, with a signal at *m/z* 182.9 (Figure 2B), was higher. A small signal at *m/z* 168.98, which was detected by both techniques, suggests the sulfation of the fucose residue at C-3 because a corresponding fragment ion (also very small) was observed for the sulfated at C-3 α-l-Fuc*p* standard under ESIMS/MS [35]. Additionally, a fragment ion from the loss of a water molecule at *m/z *225.01 was also low. No signal was observed at *m/z* 168.98 in the same MS/MS experiment using monosulfated fucose from the fucoidan of *F. evanescens* [1,37], and the signal at *m/z *225.01 was increased. Thus, the additional fragmentation and desulfation of oligosaccharides due to the ion source of the mass spectrometer could be explained by the tendency of 3-sulfated fucose residues to decompose near the sulfate groups, which is likely assisted by the cleavage of predominantly 4-linked galactose residues, as the fragment ions were “mixed” ones.

**Figure 3:**
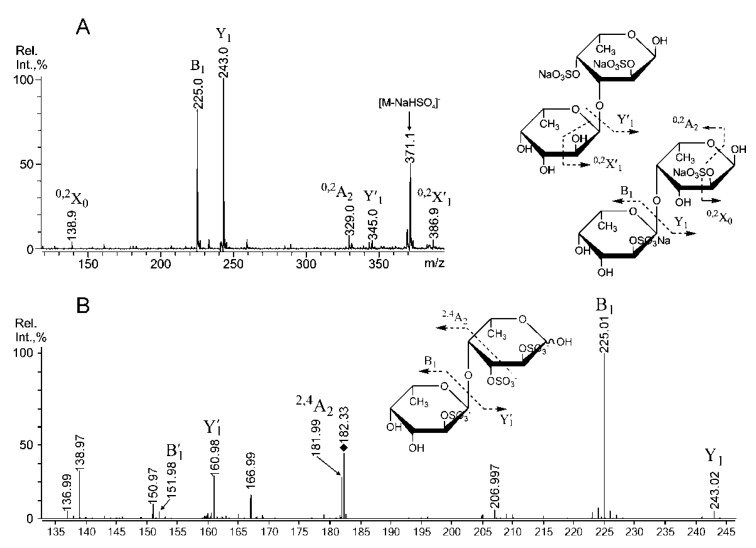
Negative-ion tandem MALDI-TOFMS of [Fuc_2_(SO_3_Na)_2_ − Na]^−^ at *m/z* 491.00 (**A**), CID ESIMS/MS of [Fuc_2_(SO_3_)_3_]^3−^ at *m/z* 182.323 (**B**).

Both tandem MALDI-TOFMS (Figure 3A) and ESIMS (not shown) of [Fuc_2_(SO_3_Na)_2_ − Na]^−^ at *m/z* 491.00 showed a similar composition of the most abundant ions compared to MS/MS of the same parent ion, which was derived from 1,3-α-l-fucan from *S. cichorioides* [2].

These data suggest that the disaccharide was mainly composed of (1→3)-linked α-l-Fuc*p *residues (46% due to methylation analysis data; Table 2). The lack of an ion at *m/z* 326.9, which corresponds to a disulfated, non-reducing α-l-Fuc*p* residue, suggests that sulfates were likely cleaved from the C-4 position of that residue and/or C-4 was the branching point (12%; Table 2) from the main chain, which was composed of 3-linked α-l-Fuc*p* residues. The high intensity of an in-source fragment at *m/z* 371.1, likely from the loss of a sulfate [35], combined with the high intensity of the Y_1_ ion at *m/z* 243.0 suggested that a sulfate group occupied the C-2 position of the reducing sugar. The high intensity of the B_1_ fragment ion at *m/z* 225.0 suggested sulfation at the C-2 position of the non-reducing sugar [1,2,38]. MS/MS also contained minor signals, including ^0,2^X_0_ at *m/z* 138.9, ^0,2^A_2_ at *m/z* 329.0 and ^0,2^X′_1_ at *m/z* 386.9. These signals were high when observed in MS/MS of the same disulfated fucobiose, derived from fucan of *F. evanescens *[1], which is rich in 4-linked α-l-Fuc*p* residues. However, only ^0,2^X_0_ was detected in ESIMS/MS. The presence of the fragment ion Y′_1_ suggests that the reducing sugar could be 2,4-disulfated, which is not the case for *F. evanescens*, but it is highly sulfated in the 3-linked fucan from *S. cichorioides *[2]. It is important to note that some signals from the cross-ring cleavages were significantly higher when analyzed using ESIMS/MS, likely due to the more intensive secondary cleavages/unknown decomposition pathways in CID ESIMS/MS. These signals included an ^0,2^X_0_ signal at *m/z* 138.9 and an ^0,2^A_1_ at *m/z* 182.9, which were not detected in tandem MALDI-TOFMS. Additionally, no ^0,3^X/A fragments were detected using either method, although some were registered for disaccharide from *S. cichorioides* [2]. Tandem ESIMS of the ion [Fuc_2_(SO_3_)_3_]^3−^ at *m/z* 182.323 (Figure 3B) revealed an intensive Y-type ion at *m/z* 160.98 for the cleavage of a doubly sulfated reducing fucose residue. A non-reducing fucose residue was preferably sulfated at C-2 (B_1_-type ion at *m/z* 225.01 [35]), and a doubly sulfated B-type ion had low intensity. ESIMS/MS also contained a signal at *m/z* 181.99, which determined to be an ^2,4^A_2_-type ion that likely arose, due to sulfation at C-3 and C-2 of the reducing fucose residue, since the intensity of this ion is too high for ^0,2^X_1_-type signal, which was not detected in ESIMS/MS (and was low in MALDIMS/MS, Figure 3A) of a doubly-sulfated variant (not shown). Additionally, it was recently shown that the sulfation of the non-reducing fucose residue at C-2 suppresses the intensity of this ion [1].

**Figure 4:**
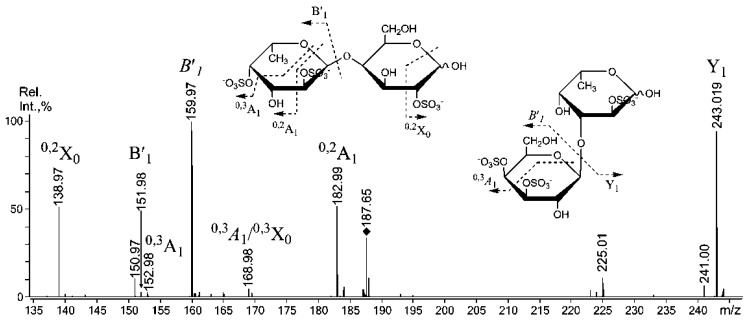
Negative-ion ESIMS/MS of [GalFuc(SO_3_)]^3−^ at *m/z *187.65.

Tandem MALDI-TOFMS (not shown) of galactose-containing disaccharides (ions at *m/z* 405.0 and 507.03; Table 3) revealed that galactose residues were preferably located at the non-reducing end. These data are supported by the methylation analysis (8 percent of Gal residues are non-reducing; Table 2). Similar results were obtained for *Costaria costata* [13] and *Saccharina latissima* by mass spectrometric method and ^13^C NMR analyses, respectively. It was shown that galactose residues could terminate chains, which was composed of 3-linked α-l-Fuc*p *residues. Tandem ESIMS analysis of the ion at *m/z* of [GalFuc(SO_3_)]^3−^ at *m/z *187.654 (Figure 4) revealed additional data.

**Table 3:** Proposed structural features of some oligosaccharides, obtained from fucoidan **SmF3** of brown alga *S. mcclurei* by autohydrolysis.

*m/z*		Composition/structural features of mono- and oligosaccharides
MALDI [M * − Na]^−^	ESI [M − nNa]^n−^	
243.00	243.016	Fuc(4SO_3_^−^), Fuc(2SO_3_^−^), Fuc(3SO_3_^−^)
-	259.012	Gal(4(6)SO_3_^−^), Gal(2SO_3_^−^)
389.10	389.077	[Fuc_2_(SO_3_)]^−^
405.10	405.070	Gal(2SO_3_^−^)-(1,3)-Fuc
491.04	234.010^2−^	Fuc(2SO_3_^−^)-(1,3)-Fuc(2SO_3_^−^) Fuc-(1,3)-Fuc(2,4SO_3_^−^) Fuc(2SO_3_^−^)-(1,4)-Fuc(2SO_3_^−^) **
-	182.323^3−^	Fuc(2SO_3_^−^)-(1,4)-Fuc (2,3SO_3_^−^) Fuc(2,4SO_3_^−^)-(1,3)-Fuc **
-	187.654^2−^	Gal(4/6,3SO_3_^−^)-(1,3/4)-Fuc(2/3SO_3_^−^) Fuc(2,4SO_3_^−^)-(1,4)-Gal(2SO_3_^−^)
507.03	242.008	Gal(2SO_3_^−^)-(1,3)-Fuc(2SO_3_^−^) Gal(2SO_3_^−^)-(1,3)-Fuc(2SO_3_^−^) **
535.15	535.136	[Fuc_3_(SO_3_)]^−^
-	231.007	[Fuc_3_(SO_3_)]^3−^
551.15	-	Gal(2SO_3_^−^)-(1,3)-Fuc-(1,3)-Fuc
-	236.341	[Fuc_2_Gal(SO_3_)_3_]^3−^
653.05	315.040^2−^	[Fuc_2_Gal(SO_3_Na)_2_ − Na]^−^
681.16	-	[Fuc_4_(SO_3_)]^−^
697.15	-	[Fuc_3_Gal(SO_3_)]^−^
783.04	380.074^2−^	[Fuc_4_(SO_3_Na)_2_ − Na]^−^
799.03	388.070^2−^	[Fuc_3_Gal(SO_3_Na)_2_ − Na]^−^
-	285.028^3−^	Gal(2SO_3_^−^)-(1,3)-Fuc-(1,3)-Fuc(SO_3_^−^)-(1,3)-Fuc(SO_3_^−^) Gal(4/6,2SO_3_^−^)-(1,3)-Fuc-(1,3)-Fuc-(1,3)-Fuc(2SO_3_^−^) Fuc-(1,4)-Gal(3SO_3_^−^)-(1,3)-Fuc(2SO_3_^−^)-(1,3)-Fuc(2SO_3_^−^)
827.13	-	[Fuc_5_(SO_3_)]^−^
843.12	-	[Fuc_4_Gal(SO_3_)]^−^
859.10	-	[Fuc_3_Gal_2_(SO_3_)]^−^
944.97	460.094^2−^	[Fuc_4_Gal(SO_3_Na)_2_ − Na]^−^
960.95	-	[Fuc(2SO_3_Na)-(1,4)-Gal-(1,3)-Fuc(2SO_3_Na)-(1,4)-Gal-(1,3)-Fuc − Na]^−^ [Fuc(2SO_3_Na)-(1,4)-Gal(2SO_3_Na)-(1,3)-Fuc-(1,4)-Gal-(1,3)-Fuc − Na]^−^ [Gal(2SO_3_Na)-(1,3)-Fuc(2SO_3_Na)-(1,4)-Gal-(1,3)-Fuc-(1,3)-Fuc − Na]^−^ [Gal(4SO_3_Na)-(1,3)-Fuc-(1,4)-Gal-(1,3)-Fuc(4SO_3_Na)-(1,3)-Fuc − Na]^−^
-	339.051^3−^	[Fuc_3_Gal_2_(SO_3_)_3_]^3−^
989.05	-	[Fuc_5_Gal(SO_3_)^−^
1005.04	-	[Fuc_4_Gal_2_(SO_3_)]^−^
1062.79	338.551	[Fuc_3_Gal_2_(SO_3_Na)_3_ − Na]^−^
1090.87	-	[Fuc_5_Gal(SO_3_Na)_2_ − Na]^−^
1106.87	541.065	[Fuc_4_Gal_2_(SO_3_Na)_2_ − Na]^−^

* M represents sodium salt of sulfated oligosaccharides; ** Characteristic fragment ions, suggesting represented linkage type have low intensity.

**Figure 5:**
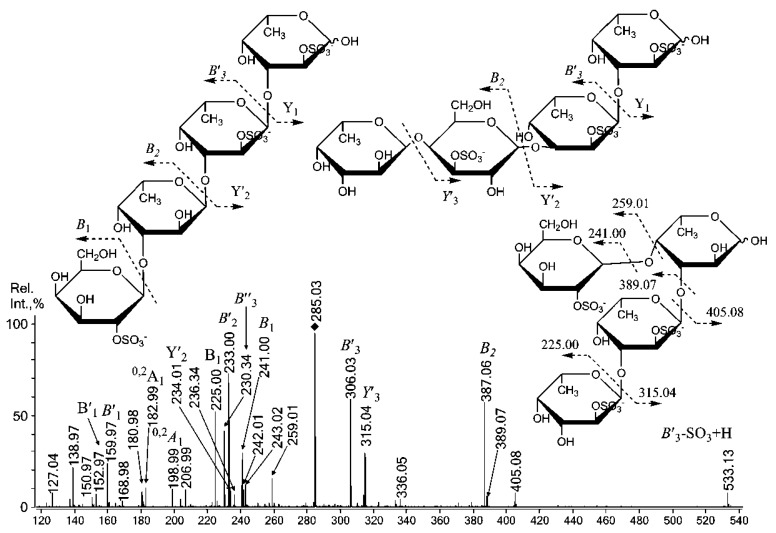
Negative-ion ESIMS/MS of [Fuc_3_Gal(SO_3_)]^3−^ at *m/z* 285.028*.*

Indeed, the intensity of the *B*′_1_ ion (the italic font indicates that ion is mixed, and “′”indicates that it is doubly sulfated) at *m/z* 159.97 was highest, indicating that the structural variant with a doubly sulfated non-reducing galactose was prevalent. However, the other structural variant with non-reducing fucose that was sulfated at C-4 [35] displayed a signal at *m/z* 182.99 (^0,2^A_1_), which is rather high. However, both B-type ions at *m/z* 225.01 and 151.98 were low. Hence, a similar signal at *m/z* 199 (^0,2^A_1_) should have been detected, but none was found. This result could be explained by sulfation at the C-3 position of the non-reducing galactose residue, along with sulfation at the C-4/C-6, which is indicated by the ^0,3^A_1_-signal at *m/z* 168.98, which also suggests the sulfation of the reducing fucose residue at C-3 [35]. The ESIMS/MS spectrum (Figure 5) of the triply sulfated tetrasaccharide ion [Fuc_3_Gal(SO_3_)_3_]^3−^ at *m/z* 285.028 suggested the presence of at least three structural variants. 

For the first time, only due to MS/MS data of the sulfated fucooligosaccharides one is allowed to suggest structural variant with branched structure: the lack of the ions for the successively cleaved fucose residues (no diagnostic ions of B-type, such as at *m/z* 298.04^2−^ for the cleavage of a fucotriose and/or 225.01^2−^ for the cleavage of a doubly-sulfated fucobiose with loss of water as well as their singly-sulfated variants). Structural variant with the reducing sulfated galactose (F-F-F-G), suggested by *Y*_1_ at *m/z* 259.01 does not exist, due to the mentioned above. The F-F-G-F variant also does not exist either, as no *Y*″_2_ signal was observed at *m/z *187.66. Surprisingly, internal galactose residues did not show any cross-ring cleavages, and 4- and 3-linked Gal residues were not detected by methylation analysis (Table 2). The lack of cross-ring cleavages could be explained by sulfation at the C-3 position of Gal residues [35]. It is possible that sulfate at C-3 is more stable in selected conditions.

**Figure 6:**
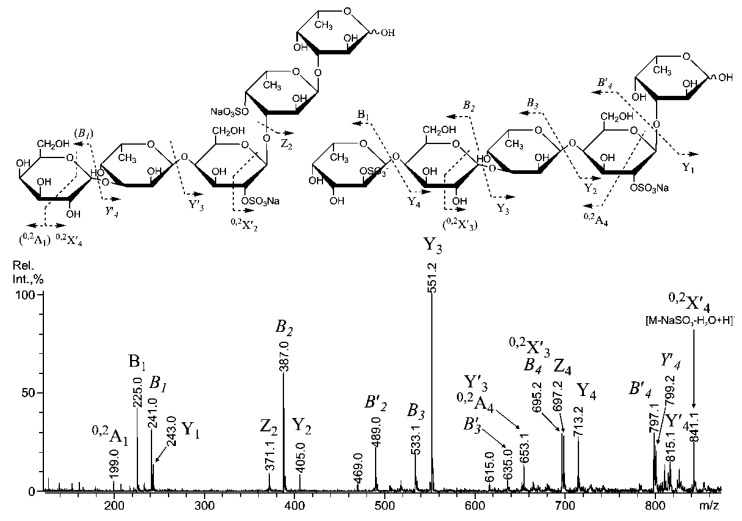
Negative-ion tandem MALDI-TOFMS of [Fuc_3_Gal_2_(SO_3_Na)_2_ − Na]^−^ ion at *m/z* 960.95. Fragment ions, enclosed in brackets are corresponding to the (doubly) sulfated monosaccharide residues, sulfate groups of which are not shown to minimize drawing.

The most interesting tandem MALDI-TOFMS of mixed oligosaccharides that contained galactose and fucose ([Fuc_3_Gal_2_(SO_3_Na)_2_ − Na]^−^) was found at *m/z *960.95 (Figure 6).

The selected ion could be a fragment ion from the desulfation of a triply sulfated ion [Fuc_3_Gal_2_(SO_3_)_3_]^3−^ that was detected by ESIMS at *m/z* 339.051. However, ESIMS/MS was uninformative due to the low intensity of the parent ion. A tandem MALDI-TOFMS was relatively simple instead. These data suggested at least four structural variants of the selected ion (Table 3). The doubly sulfated variants of some ions, produced by glycosidic bonds cleavages (as mentioned above) and cross-ring cleavages, are marked by an apostrophe (“′”) to minimize drawing, as a glycosidic bond is likely to be cleaved near the sulfated residue [33]. The complete series of Y- and B-type ions proposed the presence of unique structural variants of the selected ion: Fuc-(1→4)-Gal-(1→3)-Fuc-(1→4)-Gal-(1→3)-Fuc and Gal-(1→3)-Fuc-(1→4)-Gal-(1→3)-Fuc-(1→3)-Fuc. These variants were randomly sulfated (due to the random loss of sulfates) at positions C-2 or C-4 of both Gal and Fuc residues. The type of Gal residue linkage was mainly determined by methylation data because cross-ring cleavages from Gal residues overlap with fragment ions from glycosidic bonds cleavages (Figure 6).

Thus, the structural features of the fucoidan **SmF3** were predominantly determined by a mass spectrometric analysis of the LMW oligosaccharide fragments that were obtained by autohydrolysis. These analyses indicated the presence of mixed oligosaccharides, containing galactose and fucose residues, unlike fucoidans from Laminariales [2,13], for which the autohydrolysis mixture contained only minor amounts of mixed components, including only terminal galactose. Fragments, consisting of α-l-Fuc*p* residues only with DP up to 5 and with up to 3 sulfate groups per molecule were also found, being predominantly 3-linked and, less-frequently, 4-linked, due to methylation analysis. An MS analysis revealed that both α-l-Fuc*p* and β-d-Gal*p* residues were sulfated at C-2 and/or C-4 (and some at C-6 of β-d-Gal*p*, due to the comparison of ^13^C NMR data of sulfated and desulfated fucoidans) and, possibly, at the C-3 position of terminal β-d-Gal*p *and 4-linked α-l-Fuc*p* residues, as the corresponding fragments were found and characterized: Fuc(2SO_3_^−^)-(1→3)-Fuc(2SO_3_^−^), Fuc-(1→3)-Fuc(2,4SO_3_^−^), Fuc(2SO_3_^−^)-(1→4)-Fuc(2,3SO_3_^−^), Fuc(2,4SO_3_^−^)-(1→3)-Fuc; Gal(4/6,3SO_3_^−^)-(1→3/4)-Fuc(2/3SO_3_^−^), and Fuc(2,4SO_3_^−^)-(1→4)-Gal(2SO_3_^−^). The predominant type of β-d-Gal*p* residues linkage was identified via methylation analysis to be (1→4), and in this case, the sulfate occupied position C-3 of β-d-Gal*p* residues, as the following fragments were detected: Fuc-(1→4)-Gal(3SO_3_^−^)-(1→3)-Fuc(2SO_3_^−^)-(1→3)-Fuc(2SO_3_^−^). Characteristic cross-ring cleavages to 4-linked galactose residues were not detected, likely due to sulfation at C-3. Further analysis of mixed oligosaccharides with higher DP showed that the fucoidan **SmF3** contains chains of alternating sulfated α-l-Fuc*p* and β-d-Gal*p* residues, as the following structures were found: Fuc(2SO_3_^−^)-(1→4)-Gal-(1→3)-Fuc(2SO_3_^−^)-(1→4)-Gal-(1→3)-Fuc, Gal(2SO_3_^−^)-(1→3)-Fuc(2SO_3_^−^)-(1→4)-Gal-(1→3)-Fuc-(1→3)-Fuc. Successively linked galactose residues were not observed, unlike fucoidans from Laminariales, which contain galactan chains [2,13,27,28].

Our data suggest that oligosaccharides, obtained by autohydrolysis, were formed from branches and the most labile part of native fucoidan core in selective condition. The pellet, which is resistant to autohydrolysis, likely contained fragments of the main chain, including 6-linked galactose on the reducing end, as it was not found in low-molecular-weight fraction **SmF3-AH**. Therefore, we assume that 1,6-links are stable in autohydrolysis conditions.

Due to random desulfation during autohydrolysis and/or in the ion source of mass spectrometer, some residues were detected as free of sulfates. However, **SmF3** is a heavily-sulfated polysaccharide (35%), Thus, it is possible that almost all of the free OH-groups on native polysaccharide are sulfated (considering spatial restrictions) because ^13^C NMR did not detect any acetyl groups.

### 2.4. Cytotoxicity and Antitumor Activity of SmF1, SmF2, SmF3, and SmF3-DS

**Figure 7:**
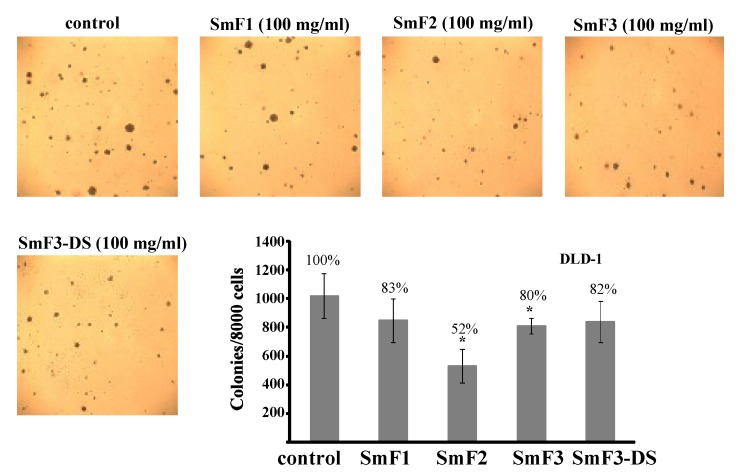
The inhibitory effects of fucoidans from brown seaweeds *S. mcclurei* on colony formation in human colon cancer cells DLD-1 comparing untreated control cells. Cells (2.4 × 10^4^/mL) treated with/without fucoidans (100 μg/mL) were exposed in 1 mL of 0.3% BME’s agar containing 10% FBS. The culture was maintained at 37 °C in a 5% CO_2_ atmosphere for three weeks. The colonies were counted under a microscope with the aid of the ImageJ soft ware program. Data are represented as the means SD of the number of colonies are determined from three independent experiments.

Fucoidans possess a multitude of biological activities. We examined the effect of sulfated polysaccharides from *S. mcclurei* on the cytotoxicity of DLD-1 human colon cancer cells using 3-(4,5-dimethylthiazol-2-yl)-5-(3-carboxymethoxyphenyl)-2-(4-sulfophenyl)-2*H*-tetrazolium, inner salt (MTS assay). Fucoidans did not show any significant cytotoxicity after treatment for 24 and 48 h at 1 to 200 μg/mL (supplementary data). These results confirm data obtained in previous studies. The sulfated polysaccharides from other species of brown algae were found to be nontoxic against JB6 Cl41 (epidermal mouse cells), Vero (African green monkey kidney), MCF-10A (human epithelial cells), MCF-7 (human breast cancer cells) and other cells [39,40,41]. Our results indicate that these polysaccharides are not cytotoxic towards DLD-1 human colon cancer cells at concentrations from 1 to 200 μg/mL. Next, we determined whether purified polysaccharides inhibited colony formation (soft agar method) in human colon cancer DLD-1 cells. This assay is a well-established model for studying the potential of antitumor agents. DLD-1 colon cancer cells were treated with 100 μg/mL fucoidan in a soft agar matrix, and the cells were incubated at 37 °C in a 5% CO_2_ incubator for three weeks. The tested polysaccharides had antitumor activity on DLD-1 cells at 100 μg/mL. **SmF1**, **SmF2**, **SmF3**, and **SmF3-DS** inhibited the colony formation of DLD-1 colon cancer cells by 17, 48, 20, and 18%, respectively (Figure 7).

According our results, the degree of sulfation did not play a role in the inhibition of colony formation. After desulfation, no change was observed in the ability of fucoidan **SmF3** (35% sulfate content) to inhibit colony formation. Additionally, the extent of colony formation inhibition in DLD-1 colon cancer cells by **SmF2** (25.7% sulfate content) was higher than that of **SmF3**. Thus, we show that fucoidans from *S. mcclurei* (**SmF1**, **SmF2**, and **SmF3**) possess anticancer activity against DLD-1 colon cancer cells. Probably, the presence of alternating sulfated 1,3-linked α-l-Fuc*p* and 1,4-linked β-d-Gal*p* residues is important for this activity.

Taken together, these data are the first to demonstrate that fucoidans isolated from *S. mcclurei* have antitumor activity against the DLD-1 colon cancer cells.

## 3. Experimental Section

### 3.1. Materials

Organic solvents, inorganic acids and salts, sodium hydroxide, and trifluoroacetic acid (TFA) were commercially available (Laverna, Russian Federation). Standards (mannose, rhamnose, glucose, galactose, and xylose) were purchased from Sigma (USA). RPMI-1640 medium, fetal bovine serum (FBS), l-glutamine, penicillin-streptomycin, and gentamicin were purchased from Biolot (Russia). The CellTiter 96 nonradioactive cell proliferation assay kit was purchased from Promega (USA). The sorbents used for chromatography were Polychrome-1 (Reakhim, Russian Federation) and Macro-Prep DEAE (Bio-Rad, USA).

All MS experiments were performed using ultra pure water from Direct-Q 3 equipment (Millipore, USA). Arabinoosazone (phenylosazone of d-arabinose) was synthesized as previously described [42].

The samples of the alga, *Sargassum mcclurei *(Setchell, 1933), were collected in June 2010 from Nhatrang Bay (Socialist Republic of Vietnam) aboard the ship, “Academic Oparin”. The collected algae were identified [43], washed in seawater, and then in distilled water to remove mud, sand, salt and foreign substances. Then, the algae were air-dried in well-ventilated place without direct sunlight at room temperature for three days and milled to powder (particle size = 3–4 mm). The dried powder algae (620 g) were pretreated with EtOH:CHCl_3_:H_2_O (89:1:10) (w/v = 1:10) for 10 days to remove pigments and other low-molecular weight compounds. Then, the algae were air-dried in well-ventilated place at room temperature for two days.

### 3.2. Instruments

NMR spectra were obtained on an Avance DPX-500 NMR spectrometer (Bruker, Germany) resonating at 75.5 MHz at 35 °C. The sample concentration was 20 mg of polysaccharide/mL of D_2_O for ^1^H and ^13^C experiments.

MALDI-TOFMS spectra were recorded using an Ultra Flex III MALDI-TOF/TOF mass spectrometer (Bruker, Germany) with a nitrogen laser (337 nm), reflector and the potential LIFT tandem modes of operation.

ESIMS spectra were recorded using an ESI Q-TOF mass spectrometer (Agilent 6510 LC Q-TOF, USA) with a dual electrospray-ionization source.

### 3.3. General Methods

#### 3.3.1. Analytical Procedures

Total carbohydrates were quantified using the phenol-sulfuric acid method [44]. Monosaccharides composition was determined after polysaccharide hydrolysis by 2 M TFA (6 h, 100 °C) by HPLC using column ISA-07/S2504 (0.4 × 25 cm, Shimadzu), a bicinhoninate assay, and a C-R2 AX integrating system (Shimadzu, Japan). The protein and polyphenol contents were determined using the Bradford [45] and Folin-Ciocalteau [46] methods, respectively. Sulfate groups were determined using the BaCl_2_ gelatin method [47].

#### 3.3.2. Mass Spectrometric Analysis

MALDI-TOFMS. Analyses were performed in a negative-ion mode using arabinoosazone as a matrix, which was synthesized as previously described [42]. The matrix solution was prepared by mixing 10 mg/mL of arabinoosazone solution in ethanol and 10 mg/mL of l-fucose in water to reduce in-source fragmentation [48]. The sample solution (0.01 mg/mL in water) was prepared from lyophilized samples. The “thin layer” technique was used to prepare the samples. Briefly, 1 μL of matrix solution was applied to a stainless steel plate and allowed to dry. Then, 1 μL of sample solution was applied as the second layer and air-dried. The instrument was precalibrated using matrix and angiotensin-II (Sigma, USA) ion signals.

ESIMS. Spectra were acquired in both positive and negative-ion modes, and pre-calibration was performed using a standard “HP-mix”. The capillary voltage was set to 4000 V, and the drying gas temperature was 325 °C. The fragmentor voltage was set to 160 V. The isolation window for MS/MS experiments was set to 1.3 mass units for singly charged ions and 4 mass units for multiply charged ions. The collision energy was optimized between 10 and 45 V to reach the most abundant intensity of fragment ions. Dried samples were dissolved in 1:1 acetonitrile-water (the concentration of the sample was approximate 0.01 mg/mL), and they were introduced into the mass spectrometer at a flow rate of 5 μL/min using a syringe pump (KD Scientific, USA).

#### 3.3.3. Water-Soluble Polysaccharide Extraction

**Figure 8:**
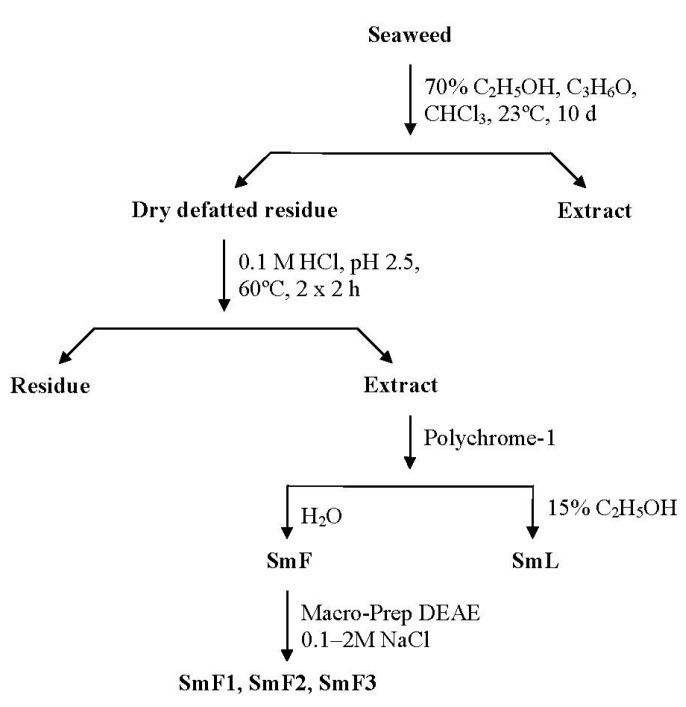
Scheme of isolation and separation of water-soluble polysaccharides from brown alga *S. mcclurei*.

Samples of defatted, dried, and powdered algal fronds (500 g) were extracted twice with 0.1 M HCl (5 L) for 2 h at 60 °C. The extracts were collected by centrifugation, combined, concentrated under vacuum and dialyzed against distilled water using a 5 kDa cut-off membrane for 48 h. A schematic of the methods used to isolate and separate the water-soluble polysaccharides is shown in Figure 8.

#### 3.3.4. Hydrophobic Chromatography

Extracts containing water-soluble polysaccharides were applied to a Polychrome-1 (Reakhim, Russia) column (Figure 8). The crude fucoidan fraction (**SmF**) was eluted with water, and the laminaran fraction (**SmL**) was eluted with 15% aqueous ethanol until the disappearance in the eluent of positive reaction for carbohydrates by the phenol-sulfuric acid method [44]. The corresponding polysaccharide fractions were concentrated under a vacuum and lyophilized.

#### 3.3.5. Anion-Exchange Chromatography

A solution of crude fucoidan **SmF** in 0.1 M NaCl (0.5 g in 10 mL) was applied to a Macro-prep DEAE (Bio-Rad, USA) column (Cl^−^ form, 2.5 × 9 cm) that was equilibrated with 0.1 M NaCl (Figure 8). The column was then successively eluted with a linear gradient of NaCl (from 0.1 to 2 M) until the disappearance in the eluent of positive reaction for carbohydrates by the phenol-sulfuric acid method [44]. The corresponding polysaccharide fractions were concentrated under a vacuum, dialyzed against distilled water using a 5 kDa cut-off membrane for 48 h, and lyophilized.

#### 3.3.6. Desulfation of Fucoidan

An 10 mL aliquot of the fucoidan **SmF3** (20 mg/mL in water) was passed through a Timberlite CG-120 column (200–400 mesh, H^+^-form, 1 × 5 cm, Serva, Germany) with H_2_O. After neutralization with pyridine (0.5 mL), the solution was lyophilized. The resulting pyridinium salt of the fucoidan was dissolved in DMSO (18 mL) and pyridine (0.6 mL), and it was then incubated at 100 °C for 3 h. The solution was exhaustively dialyzed against distilled water using a 5 kDa cut-off membrane for 48 h and lyophilized to obtain desulfated fucoidan **SmF3-DS**.

#### 3.3.7. Methylation of Fucoidan

The sample of desulfated fucoidan **SmF3-DS** (2 mg) was solubilized in DMSO (1 mL), and powdered NaOH (100 mg) was added to the solution, followed by MeI (0.2 mL). The mixture was stirred for 20 min, and NaOH and MeI were added again. This mixture was stirred periodically for 1 h, and it was then cooled on ice. The reaction was terminated by the addition of 1 mL of water. The excess MeI was removed by concentration under a vacuum, and the resulting solution was passed through a Silica gel 100 С18 column (2.5 × 0.7 cm, Sigma, USA). Methylated fucoidan was eluted with 50% MeOH, concentrated under a vacuum, and hydrolyzed with 2 M TFA at 100 °C for 6 h. TFA was neutralized with aqueous NH_3_ (5%), and the resulting solution was concentrated and lyophilized under a vacuum. Then, the resulting monosaccharides were reduced with NaBH_4_ and acetylated with Ac_2_O in pyridine. Partially methylated alditol acetates were analyzed by GLC-MS, as previously described [26].

#### 3.3.8. Depolymerization of Fucoidan by Autohydrolysis

Fucoidan preparation **SmF3** was submitted to autohydrolysis for depolymerization (“autohydrolysis” is used here to denote acidic polysaccharide hydrolysis under very mild conditions using the –SO_3_H groups of the compound as the acid source). A 20 mL aliquot of the fucoidan **SmF3** (5 mg/mL in water) was changed to the H^+^-form using a minicolumn for cation exchange (Timberlite CG-120, 200–400 mesh, Serva, Germany) and left for 48 h at 37 °C. The mixture was then neutralized with aqueous NH_3_ (5%) and lyophilized. The low molecular weight fraction, **SmF3-AH**, was obtained by fractionation in H_2_O/EtOH (1:5 w/w). The supernatant was freeze-dried. The yield of the **SmF3-AH** fraction from the **SmF3** was 85%. The monosaccharide composition of the **SmF3-AH** (mol%) was as follows: Fuc, 68.5 and Gal, 31.5. The monosaccharide composition of the pellet, which was resistant to autohydrolysis, (mol%) was as follows: Fuc, 85.3 and Gal, 14.7. The dried preparation of **SmF3-AH** was dissolved in water to 0.1 mg/mL for MALDIMS or dissolved in 1:1 CH_3_CN–H_2_O to 0.05 mg/mL for ESIMS analysis.

### 3.4. Anticancer Activity

#### 3.4.1. Cell Culture

The DLD-1 (ATCC # CCL-221™) human colon cancer cell line was grown as a monolayer in RPMI-1640 supplemented with 10% (v/v) heat-inactivated FBS, 2 mM l-glutamine, and 1% penicillin-streptomycin in humidified atmosphere containing 5% CO_2_.

#### 3.4.2. Cell Cytotoxicity Assay

To estimate cell cytotoxicity, cells were seeded (3 × 10^4^) in 96-well plates in 200 μL of 10% FBS RPMI-1640 at 37 °C in a 5% CO_2_ incubator. After 24 h, the medium was removed and replaced with fresh medium containing different concentrations (50, 100, or 200 μg/mL) of the polysaccharides, and the cells were incubated for an additional 24 and 48 h at 37 °C in a 5% CO_2_ incubator. After incubation, 3-(4,5-dimethylthiazol-2-yl)-5-(3-carboxymethoxyphenyl)-2-(4-sulfophenyl)-2*H*-tetrazolium, inner salt (MTS reagent) (10 μL) was added to each well, and the cells were incubated for 4 h at 37 °C and 5% CO_2_. The absorbance was measured at 490/630 nm.

#### 3.4.3. Soft Agar Clonogenic Assay

A soft agar assay was performed using human colon cancer cells (DLD-1). Briefly, the cells (2.4 × 10^4^/mL) were treated and with fucoidans (100 μg/mL) or the vehicle and grown in 1 mL of 0.3% basal medium Eagle’s agar containing 10% FBS, as described [49]. The culture was maintained at 37 °C in a 5% CO_2_ incubator for 3 weeks, and the resulting cell colonies were scored using a microscope and the ImageJ computer software program.

#### 3.4.4. Data Analysis

All figures shown in this study are representative of at least three independent experiments with similar results. Statistical differences were evaluated using the Student’s *t*-test, and differences were considered to be significant at *p* ≤ 0.05.

## 4. Conclusions

Three different fucoidan fractions were extracted from the brown alga, *S. mcclurei*. Fucoidans **SmF1** and **SmF2** are sulfated heteropolysaccharides containing fucose, galactose, mannose, xylose and glucose. The galactofucan fraction **SmF3**, which has the highest sulfate content (35%), was thoroughly examined by ^13^C NMR (supplementary data), methylation analysis (Table 2) and mass spectrometric analysis of LMW oligosaccharide fragments, which were obtained by autohydrolysis (Table 3).

Our results indicate that the structure of the fucoidan **SmF3** can be described as follows: the main chain of the polysaccharide, possibly, was →3)-Fuc(2,4SO_3_^−^)-(1→3)-Fuc(2,4SO_3_^−^)-(1→ motif with 4-linked 3-sulfated α-l-Fuc*p* inserts and 6-linked galactose on the reducing ends. The likely branching points were 1,2,6- or 1,3,6-linked galactose (methylation analysis) and/or 1,3,4-linked fucose (MS analysis, Figure 5, on the right) residues, which could likely be glycosylated with terminal β-d-Gal*p* residues or, probably, chains of alternating sulfated α-l-Fuc*p* and β-d-Gal*p* residues. Both α-l-Fuc*p* and β-d-Gal*p* residues were sulfated at C-2 and/or C-4 (and some C-6 of β-d-Gal*p*) and potentially at the C-3 position of terminal β-d-Gal*p*, 1,4-linked β-d-Gal*p* and 1,4-linked α-l-Fuc*p* residues. The presence of alternating sulfated 1,3-linked α-l-Fuc*p* and 1,4-linked β-d-Gal*p* residues were found in galactofucans from *Sargassum* and other algae for the first time. We showed that this galactofucan inhibited colony formation in DLD-1 colon cancer cells and the degree of sulfation was not important for this effect. Fucoidans from *S. mcclurei*** SmF1** and **SmF2** also reduced colony formation in DLD-1 cells, but it was difficult to make any conclusion about the structural characteristics that were important for this because these fucoidans have a heterogeneous in monosaccharide composition. Thus, we are the first to demonstrate that the galactofucan isolated from *S. mcclurei* have antitumor activity against DLD-1 colon cancer cells.

## Supplementary Files

Supplementary File 1 Supplementary Information (PDF, 210 KB)
