# Supplementary material for: Structural Characteristics and Anticancer Activity of Fucoidan from the Brown Alga Sargassum mcclurei
Source: Mar Drugs. 2013 May 6;11(5):1456–76. doi: 10.3390/md11051456 (PMC3707154; doi:10.3390/md11051456)

Supplementary Information

Figure S1. Anion-exchange chromatography (Macro-Prep DEAE) of crude fucoidan (**SmF**) from *S. mcclurei*.

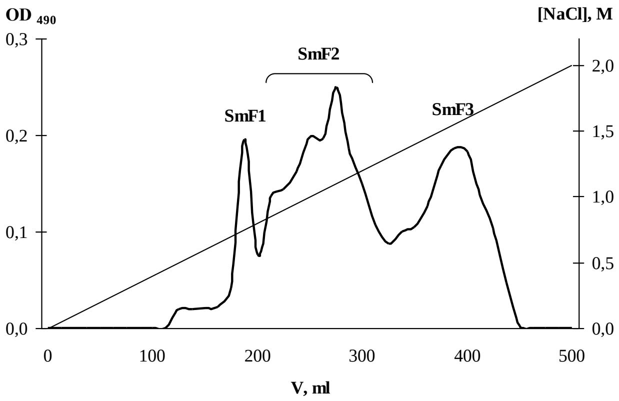

Figure S2. ^{13}C NMR spectra of native **SmF3** (**A**) and desulfated **SmF3-DS** (**B**) fucoidans from brown seaweed *S. mcclurei*.

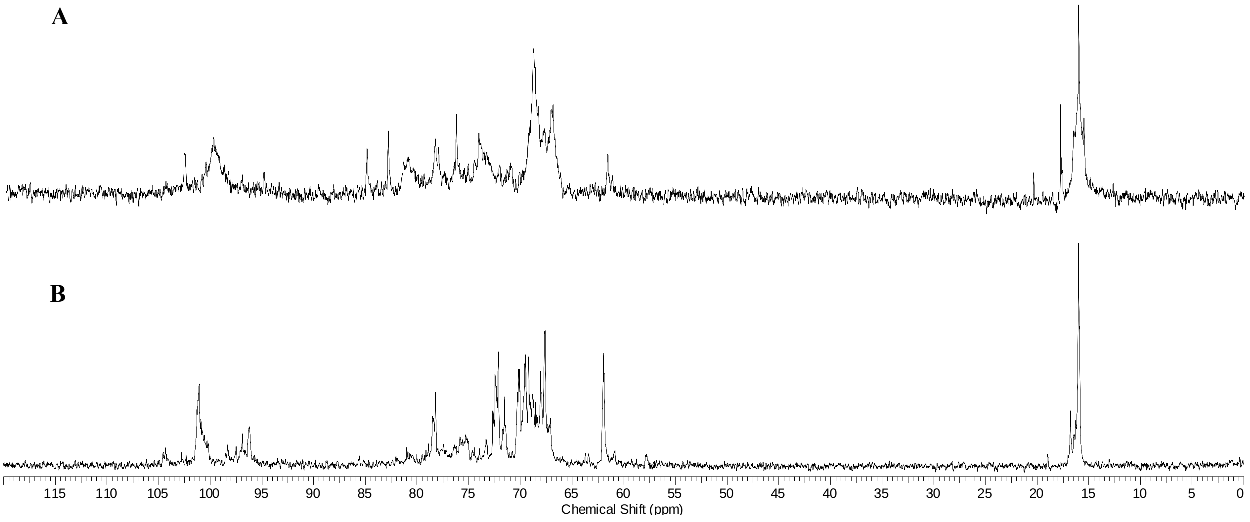

Figure S3. Negative-ion ESIMS of the **SmF3** fucoidan after autohydrolysis.

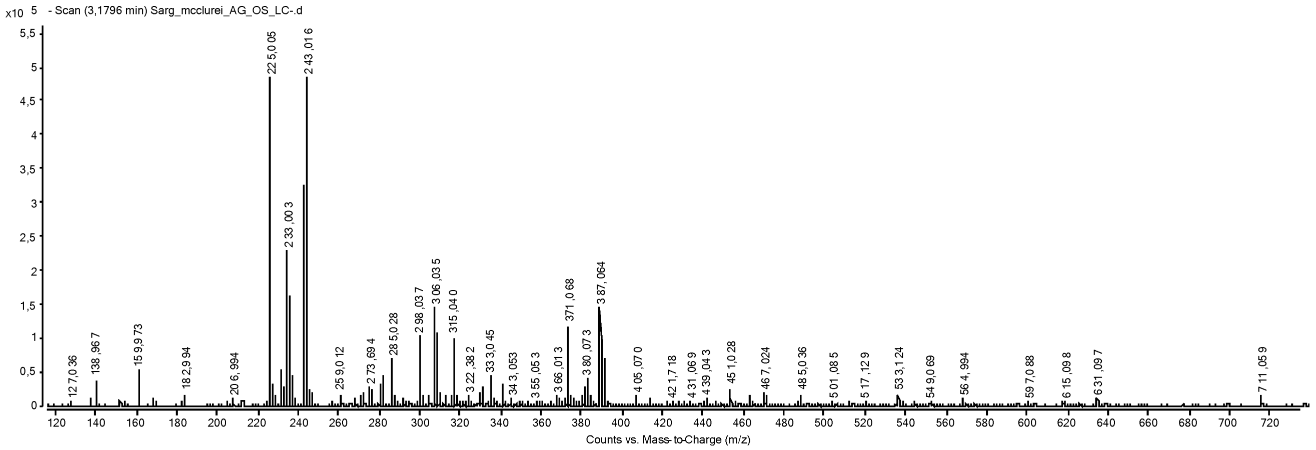

Figure S4. Negative-ion tandem CID ESIMS of the ion at *m/z* 259 (monosulfated Gal).

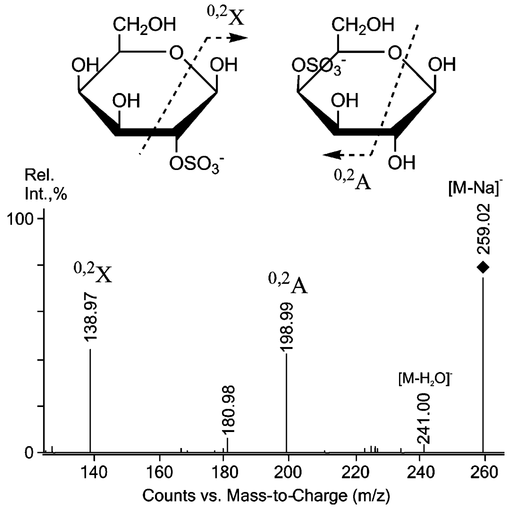

Figure S5. Cytotoxicity of the fucoidan fractions from the brown alga *Sargassum mcclurei*. DLD-1 cells (1.0 × 10^4) were incubated with investigated fucoidan fractions (200 μg/mL) for 24/48 h at 37 °C in a 5% CO_2 incubator. Compound cytotoxicity was estimated using the MTS assay. Data are represented as the mean ± SD as determined from triplicate experiments.

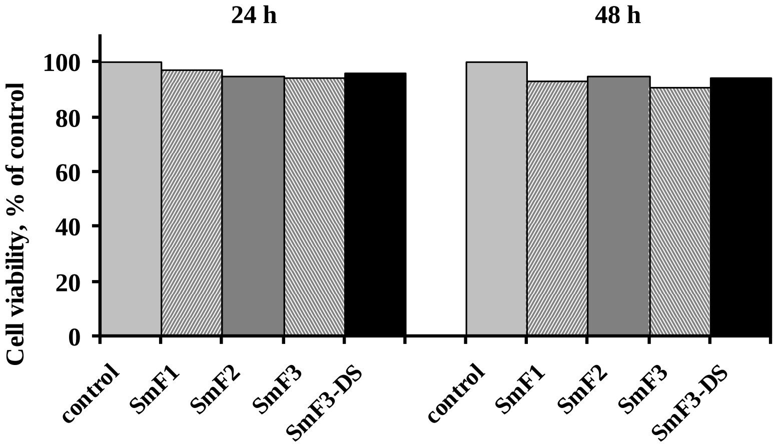

Supplement: Supplementary File 1 — Supplementary Information (PDF, 210 KB) [file marinedrugs-11-01456-s001.pdf]
